# Conservative medical management combined with follow-up multidetector computed tomography of tracheobronchial injury caused by penetrating injuries: A case report^☆^

**DOI:** 10.1016/j.tcr.2022.100710

**Published:** 2022-10-04

**Authors:** Tsuyoshi Takahashi, Tadashi Kaneko, Atsuya Hane, Asami Ito, Eiji Kawamoto, Misato Suzumura, Koki Ueda, Mari Shinoda, Atsushi Ito, Hiroshi Imai

**Affiliations:** a Emergency and Critical Care Center, Mie University Hospital, Tsu, Japan; b Department of Otorhinolaryngology, Head & Neck Surgery, Mie University Graduate School of Medicine, Tsu, Japan; c Department of Thoracic and Cardiovascular Surgery, Mie University School of Medicine, Tsu, Japan

**Keywords:** Tracheal injury, Tracheal rupture, Penetrating injury, Airway injury

## Abstract

Tracheobronchial injury (TBI) associated with penetrating injuries has various clinical symptoms and often requires urgent surgical repair. A tracheal tube and/or placement of a drainage tube combined with multidetector computed tomography (CT) could be used to manage TBI without surgical repair in eligible patients. In this case report, we describe an 86-year-old woman with subcutaneous emphysema and suspected TBI caused by three knife wounds in her neck. After tracheal intubation at a local hospital, she was transferred to our hospital. On admission, she was diagnosed with subcutaneous and mediastinal emphysema due to TBI, as well as bilateral pneumothorax. We adjusted the position of the tracheal tube to a distal location from the TBI, and placed bilateral thoracic drainage tubes by referring to the CT images taken on admission and during the follow-up. The follow-up CT images revealed healing of the TBI. She did not show any worsening of her symptoms and she was successfully extubated on day 10 of her hospital stay. On day 18, she was considered self-reliant and was transferred to her previous hospital. Based on our experience in this case, we believe that ventilation with appropriate sedation, placement of a tracheal tube, and drainage are important conservative therapies for TBI caused by penetrating injuries. CT is also useful for evaluating the status of TBI.

## Introduction

Tracheobronchial injury (TBI) caused by a penetrating injury (PI) of the neck is a potentially life-threatening condition. Current recommendations advocate surgical treatment of respiratory and/or circulatory unstable patients, such as those with unstable hemodynamics, airway management crisis, or large pneumothorax [1]. However, even stable patients with PI to neck experience TBI and/or esophageal injury necessitating surgical repair. Therefore, multiple diagnostic studies, such as computed tomography (CT) with angiography, Doppler ultrasonography, gastrointestinal endoscopy, and fluoroscopy, are necessary to establish the surgical indication in patients a PI [2]. Currently, only some patients with a neck PI associated with clinical symptoms or complications, especially TBI, can be managed without cervical exploration and surgical repair [3].

Here, we report a patient with symptomatic TBI caused by PI who was managed by conservative medical therapy, tracheal tube management, and follow-up multidetector CT. The improvement of subcutaneous emphysema, effective drainage of the pneumothorax, and absence of wound infection were important clues that prompted conservative treatment in this case. Our experience highlights the possibility that TBI caused by PI can heal spontaneously, and that follow-up CT scans can help avoid unnecessary surgical intervention.

## Case report

**Fig. 1:**
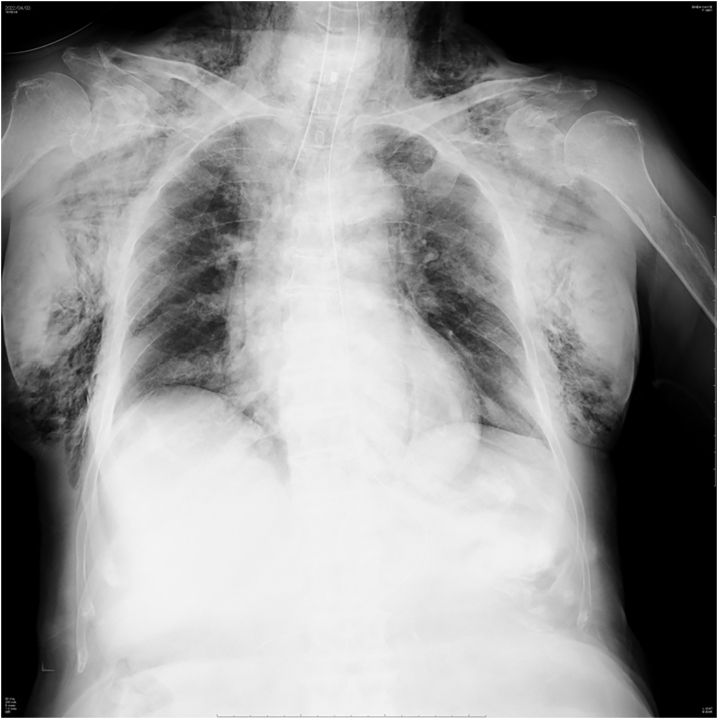
Chest radiograph on admission showing subcutaneous and mediastinal emphysema. Bilateral pneumothorax is unclear.

**Fig. 2:**
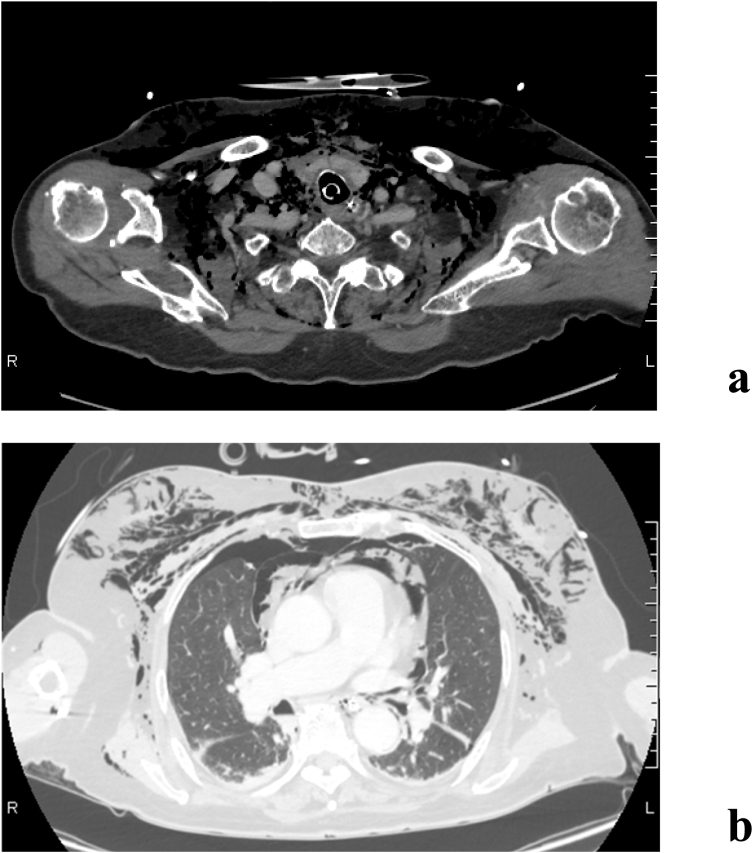
Enhanced computed tomography performed on admission showing the thyroid injury (a) and subcutaneous emphysema, mediastinal emphysema, and bilateral pneumothorax (b).

**Fig. 3:**
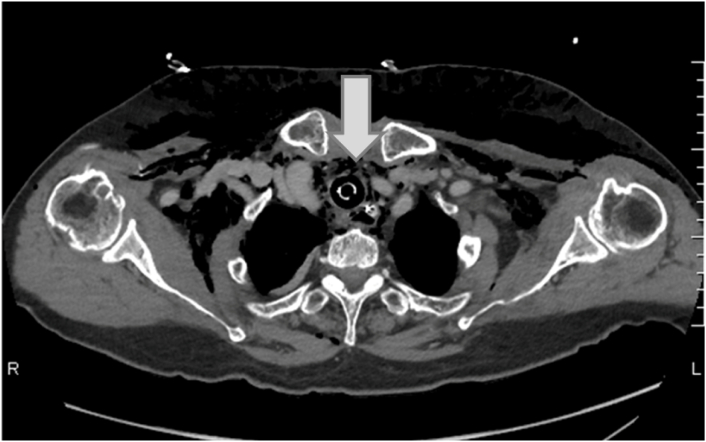
Enhanced computed tomography performed on admission showing a partial wall irregularity or a defect (arrow) in the anterior wall of the trachea.

**Fig. 4:**
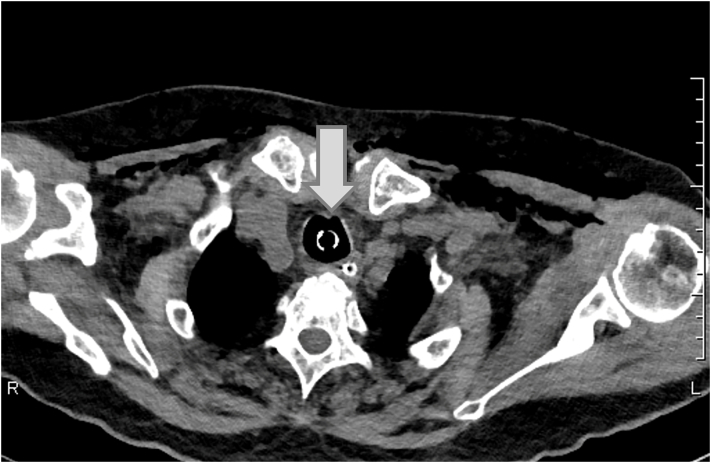
Plain computed tomography on day 4 showing anterior wall irregularity of the trachea was undetectable (arrow).

An 86-year-old woman was found in woodland with a knife in her neck and was transported to a local hospital, where subcutaneous emphysema was observed in her neck and airway injury was suspected. Because her hemodynamics were unstable, tracheal intubation was performed and she was transported to our hospital. Upon admission to our hospital, her hemodynamic and respiratory status stabilized with fluid infusion. Although the neck wound (right anterior neck, 5 cm) had already been sutured, cervical hemorrhage was observed and controlled by compression. Because we suspected cervical bleeding, X-ray and CT imaging studies were performed. The plain chest radiograph revealed subcutaneous emphysema (Fig. 1). Contrast CT images revealed massive subcutaneous emphysema, mediastinal emphysema, bilateral pneumothorax, and thyroid injury, although there were no obvious signs of vascular or esophageal injuries (Fig. 2). A partial wall irregularity of the trachea was observed on CT and an airway injury was suspected (Fig. 3). Chest drainage tubes were inserted to manage bilateral pneumothorax. Because we suspected that the balloon of the tracheal tube was situated on the airway injury, the tube was relocated to near the carina and the patient was managed with continuous sedation in an intensive care unit (ICU). Gastrointestinal endoscopy performed on day 2 of her hospital stay did not reveal any esophageal injuries, and her emphysema did not worsen from the previous day. Therefore, conservative therapy with antibiotics was continued. On day 4, her subcutaneous emphysema gradually improved. The right thoracic drain was removed on day 4 and the left thoracic drain was removed on day 5. A follow-up CT scan taken on day 5 showed that the previously observed airway wall irregularity was undetectable (Fig. 4) and extubation was planned for day 10. At the time of extubation, her trachea was checked by fibroscopy. This revealed a white scar (over 1 cm long) on the anterior wall of the trachea that was believed to be the site of injury. There was no evidence of vocal cord paralysis. Her subcutaneous emphysema did not worsen after extubation, and a CT scan on day 14 revealed minor subcutaneous emphysema. She was able to consume a solid meal on day 11, and transferred to a general ward on day 13. On day 18, she was transferred to her previous hospital.

## Discussion

Surgical interventions are often necessary for patients with symptoms suggestive of hemodynamic instability and significant airway or esophageal injury [2], [3]. However, surgical repair may have complications, and exploratory surgery may be unnecessary in some patients.

Conservative management of TBI was previously reported in postoperative cases, including those with intraoperative tracheobronchial rupture following general or thoracic surgery. In these previous cases, the patient had posterior wall tracheobronchial rupture, which was treated conservatively because the injury was small, there was no subcutaneous or mediastinal emphysema, and pneumothorax was controlled by drainage. Although prior cases showed no complications, such as infection, there are insufficient data on the outcomes of anterior wall tracheobronchial rupture [4]. Diagnostic studies of neck PI, including anterior TBI, are necessary. In a recent study of patients without dyspnea and extensive subcutaneous emphysema, 29.5 % of patients with asymptomatic perforating neck injuries require surgical interventions. However, there were insufficient data regarding TBI of the posterior wall [2]. Our patient had extensive subcutaneous and mediastinal emphysema, as well as bilateral pneumothorax, which prompted suspicion of TBI, especially of the anterior wall. However, her hypotension was treated by fluid infusion and her thyroid injury did not necessitate surgical intervention. Therefore, we continued conservative therapy with ventilation, and she did not experience further deterioration of her symptoms during hospitalization; therefore, she was successfully extubated. Some review articles have demonstrated the usefulness of CT scans, especially for assessing vascular injuries in hemodynamically stable patients [5]. Although tracheal or esophageal tract injuries are easily detected, it is sometimes not possible to rule out such injuries. Missing a tracheal or esophageal injury could be fatal, and additional tests should be considered if it is suspected [6]. Patients with small airway injuries (albeit not defined) without signs of infection could be treated conservatively. In such cases, tracheal intubation is preferably done via a laryngeal fiberoptic tube to check for and avoid widening a TBI [7] (Table 1). In this case (we thought anterior wall injury), if conservative management was failure, tracheostomy was performed to continue airway management and drainage of anterior neck, fatal complication of neck abscess should have been avoided for this elderly woman and follow-up CT scan was useful to judge that.

**Table 1:** Management for TBI by current papers.

Operative	
Indications	Hemodynamic instability Significant airway or esophageal injury [2], [3]
Conservative	
Indications	Stable hemodynamics. Appropriate tracheal tube placement. Posterior TBI rupture without emphysema was one of the indications in elective surgery cases [4]
Diagnosis	CT scan is useful for diagnosis of vascular injury [5] CT scan in not useful for diagnosis of Tracheal or esophageal tract injury [6]
Tracheal intubation	Assisted by fiberscope is preferable, not to widen TBI [7]
Outcome	29.5 % of asymptomatic perforation needs surgery [2]

TBI: tracheobronchial injury, CT: computed tomography.

Based on our experience of this case and prior literature, the management of TBI caused by PI, we (1) suggest that tracheal intubation with a fiberscope is preferable to avoid widening the TBI and to adjust the tube position away from the TBI; (2) ventilation with sedation and adjustment of the tracheal tube position are important aspects of conservative therapy; and (3) although the effective durations of conservative therapy and resting the TBI are unclear, follow-up CT is useful to monitor healing of the TBI.

## Conclusion

Conservative treatment combined with CT follow-up were successful in this patient with a symptomatic anterior TBI caused by PI.

## Funding

None.

## Patient's consent

We received the patient's consent.

## Declaration of competing interest

None.
